# Tacrolimus Increases the Effectiveness of Itraconazole and Fluconazole against *Sporothrix* spp.

**DOI:** 10.3389/fmicb.2017.01759

**Published:** 2017-09-15

**Authors:** Luana P. Borba-Santos, Leandro F. Reis de Sá, Juliene A. Ramos, Anderson M. Rodrigues, Zoilo P. de Camargo, Sonia Rozental, Antonio Ferreira-Pereira

**Affiliations:** ^1^Laboratório de Biologia Celular de Fungos, Instituto de Biofísica Carlos Chagas Filho, Universidade Federal do Rio de Janeiro Rio de Janeiro, Brazil; ^2^Laboratório de Bioquímica Microbiana, Departamento de Microbiologia Geral, Instituto de Microbiologia Paulo de Goés, Universidade Federal do Rio de Janeiro Rio de Janeiro, Brazil; ^3^Instituto Federal de Educação, Ciência e Tecnologia Rio de Janeiro, Brazil; ^4^Divisão de Biologia Celular, Departamento de Microbiologia, Universidade de São Paulo São Paulo, Brazil

**Keywords:** *Sporothrix brasiliensis*, *Sporothrix schenckii*, tacrolimus, calcineurin inhibitors, itraconazole, fluconazole

## Abstract

Calcineurin inhibitors – such as the clinically used drug tacrolimus – are active against important fungal pathogens, particularly when combined with azoles. However, tacrolimus has not been tested against sporotrichosis, an endemic subcutaneous mycosis with worldwide distribution. Here, we evaluated the activity of tacrolimus and cyclosporine A *in vitro* – as monotherapy and in combination with itraconazole or fluconazole – against yeasts of *Sporothrix brasiliensis* and *S. schenckii*, the main sporotrichosis agents in Brazil. We also analyzed the effect of tacrolimus treatment on intracellular neutral lipid levels, which typically increase after azole treatment. Tacrolimus inhibited the growth of yeasts from *S. brasiliensis* and *S. schenckii* reference isolates, with minimum inhibitory concentration (MIC) values (required for ≥50% growth inhibition) of 1 and 2 mg/L, respectively. Importantly, the combination of tacrolimus and azoles exhibited high synergy toward reference *Sporothrix* isolates. Tacrolimus combined with itraconazole significantly increased neutral lipid accumulation in *S. brasiliensis*, but not in *S. schenckii*. Clinical isolates of *S. brasiliensis* and *S. schenckii* were more sensitive to tacrolimus as monotherapy than feline-borne isolates, however, synergy between tacrolimus and azoles was only observed for feline-borne isolates. Cyclosporine A was effective against *S. brasiliensis* and *S. schenckii* as monotherapy (MIC = 1 mg/L), but exhibited no synergy with itraconazole and fluconazole. We conclude that tacrolimus has promising antifungal activity against sporotrichosis agents, and also increases the activity of the current anti-sporotrichosis therapy (itraconazole and fluconazole) in combination assays against *S. brasiliensis* feline-borne isolates.

## Introduction

The thermo-dimorphic fungi *Sporothrix brasiliensis* and *S. schenckii* are the main etiological agents of sporotrichosis (Zhang et al., 2015), an endemic disease with worldwide distribution (Chackrabarti et al., 2015) that affects 1000s of humans and cats, mainly in the Rio de Janeiro state (Gremião et al., 2017). Typically, sporotrichosis is acquired by traumatic inoculation of fungi into the skin, through cuts made by plant material containing the fungus in the filamentous form. However, the current outbreak in Rio de Janeiro is largely due to zoonotic transmission by cat scratches or bites, which inoculate the yeast form of the pathogen into the skin (Rodrigues et al., 2016). Sporotrichosis lesions in immunocompetent individuals are usually restricted to the skin, subcutaneous cellular tissue, and adjacent lymphatic vessels. However, infection can disseminate to other organs, leading to systemic disease in immunosuppressed patients (Barros et al., 2011).

The first-line antifungal therapy for both human and feline sporotrichosis is itraconazole (Kauffman et al., 2007; Gremião et al., 2015), but treatment is lengthy and considerably expensive. Also, the emergence of drug resistance is clear, with reports of failure in feline treatment (Gremião et al., 2015) and an increase in the number of strains displaying low susceptibility to itraconazole *in vitro* (Rodrigues et al., 2014a; Borba-Santos et al., 2015; Sanchotene et al., 2017). In addition, administration of itraconazole was not capable of controlling disseminated disease in a murine model of sporotrichosis by *S. brasiliensis* (Ishida et al., 2015), the species most frequently observed in the Southeast and South of Brazil (Gremião et al., 2017). Fluconazole is used as second-line therapy against sporotrichosis, when itraconazole cannot be administrated (Kauffman et al., 2007), but its antifungal activity *in vitro* is comparatively low (Marimon et al., 2008; Ottonelli-Stopiglia et al., 2014; Rodrigues et al., 2014a).

The search for new antifungal molecules is a considerable challenge in the area of fungal research, because of the similarities between fungi and their host cells, given their eukaryotic nature. Therefore, studies on new targets are important and could facilitate the development of more selective and active molecules toward fungi. A potentially interesting target for antifungal therapy is calcineurin, a Ca^2+^-calmodulin-activated protein phosphatase that, in fungi, regulates key physiological processes, including cell cycle progression, cation homeostasis, morphogenesis, and virulence (Robbins et al., 2016). Interestingly, calcineurin activity also renders fungi less sensitive to the stress induced by drug treatment (Robbins et al., 2016); thus, the pharmacological inhibition of calcineurin is a promising strategy against medically important fungi, such as *Candida* spp., *Cryptococcus neoformans*, and *Aspergillus fumigatus* (Stie and Fox, 2008).

Tacrolimus and cyclosporine A are well-known calcineurin inhibitors widely used in the clinic as immunosuppressant, in the prevention of transplant rejection (Ho et al., 1996). Tacrolimus is also used topically in the treatment of atopic dermatitis (Russel, 2002). In mammalian cells, tacrolimus (also known as FK506) binds to the FK506 binding protein (FKBP), while cyclosporine A binds to the cyclophylin, and these complexes inhibits calcineurin, preventing T lymphocyte activation, which triggers immunosuppression (Ho et al., 1996).

When combined with azoles, tacrolimus and cyclosporine A have synergistic activity *in vitro* against the pathogenic fungi *Candida* spp., *C. neoformans*, and *Aspergillus* spp. (Mody et al., 1988; Del Poeta et al., 2000; Onyewu et al., 2003; Steinbach et al., 2004; Sun et al., 2008; Uppuluri et al., 2008; Li et al., 2014; Denardi et al., 2015; Gao and Sun, 2015). Against *Candida albicans* biofilms, the synergistic effect of tacrolimus and fluconazole is due to calcineurin inhibition (Uppuluri et al., 2008). However, tacrolimus also inhibits the fungal ATP binding cassette (ABC) transporter that acts as efflux pump – exporting drugs from cells – and whose increased expression is one of the main mechanisms of resistance to azoles (Cannon et al., 2009). Del Poeta et al. (2000) showed that the synergy between fluconazole and tacrolimus against *C. neoformans* is due to ABC transporter inhibition (Del Poeta et al., 2000).

Although calcineurin inhibitors were tested in combination with azoles against a number of important fungal pathogens, no studies have addressed the combination effect of these compounds against sporotrichosis agents. Therefore, the aim of our study was to evaluate the activity of tacrolimus and cyclosporine A *in vitro*, alone and in combination with itraconazole or fluconazole, against the sporotrichosis agents *S. brasiliensis* and *S. schenckii.*

## Materials and Methods

### Fungal Isolates and Culture Conditions

The reference isolates *S. brasiliensis* CBS 133021 and *S. schenckii* CBS 132984 (genome strains 5110 and 109918, respectively (Teixeira et al., 2014)) were used in this study. *S. brasiliensis* and *S. schenckii* clinical isolates from sporotrichosis patients and feline *S. brasiliensis* isolates included in this study are shown on **Table 1**. Fungi were maintained in the filamentous (saprophytic) form at -20°C in saline solution containing 10% glucose and 10% glycerol. Prior to experiments, filamentous fungi were cultivated in potato agar dextrose agar (Difco, United States), at 35°C, for 7 days, and then converted to the pathogenic (yeast) form by cultivating conidia (10^5^ conidia/mL) in brain heart infusion broth (Difco, United States) supplemented with 2% glucose, pH 7.8, at 36°C, with orbital agitation (150 rpm) for 7 days. Yeast forms were used in all assays.

**Table 1:** Isolates of *S. brasiliensis* and *S. schenckii* used in this study.

Isolate code	Species	Source	Geographic origin	Reference
CBS 133021 (genome strain 5110)	*S. brasiliensis*	Feline sporotrichosis	Rio de Janeiro, Brazil	Teixeira et al., 2014
CBS 132984 (genome strain 109918)	*S. schenckii*	Human sporotrichosis	New York, EUA	Teixeira et al., 2014
B204	*S. brasiliensis*	Human sporotrichosis	Rio de Janeiro, Brazil	Borba-Santos et al., 2015
B428	*S. brasiliensis*	Human sporotrichosis	Rio de Janeiro, Brazil	Borba-Santos et al., 2015
B435	*S. brasiliensis*	Human sporotrichosis	Rio de Janeiro, Brazil	Borba-Santos et al., 2015
B735	*S. brasiliensis*	Human sporotrichosis	Rio de Janeiro, Brazil	Borba-Santos et al., 2015
B972	*S. brasiliensis*	Human sporotrichosis	Rio de Janeiro, Brazil	Borba-Santos et al., 2015
Ss 53	*S. brasiliensis*	Feline sporotrichosis	Rio Grande do Sul, Brazil	Rodrigues et al., 2013
Ss 172	*S. brasiliensis*	Feline sporotrichosis	Paraná, Brazil	Rodrigues et al., 2013
Ss 245	*S. brasiliensis*	Feline sporotrichosis	Rio de Janeiro, Brazil	Rodrigues et al., 2013
Ss 246	*S. brasiliensis*	Feline sporotrichosis	Rio de Janeiro, Brazil	Rodrigues et al., 2013
Ss 294	*S. brasiliensis*	Feline sporotrichosis	São Paulo, Brazil	Montenegro et al., 2014
Ss 03	*S. schenckii*	Human sporotrichosis	Rio Grande do Sul, Brazil	Rodrigues et al., 2012
Ss 42	*S. schenckii*	Human sporotrichosis	Ceará, Brazil	Rodrigues et al., 2014b
Ss 73	*S. schenckii*	Human sporotrichosis	Rio de Janeiro, Brazil	Rodrigues et al., 2013
Ss 75	*S. schenckii*	Human sporotrichosis	Rio de Janeiro, Brazil	Rodrigues et al., 2013
Ss 110	*S. schenckii*	Human sporotrichosis	Minas Gerais, Brazil	Rodrigues et al., 2013


### Compounds

Tacrolimus (Tecoland, CO., United States) was stored as 20 mM stock solutions in DMSO (kept in liquid nitrogen). Itraconazole, fluconazole, and cyclosporine A (Sigma Chemical, CO., United States) were stored as stock solutions of 6400 mg/L in DMSO (at -20°C).

### Antifungal Activity Assays

All antifungal activity assays were performed with the yeast form of *Sporothrix* isolates. The antifungal activities of itraconazole, fluconazole, tacrolimus and cyclosporine A, as monotherapy, were determined by the broth microdilution test described in the Clinical & Laboratory Standards Institute (CLSI) reference method M27-A3 (CLSI, 2008), with minor adaptation for the use with *Sporothrix* spp. yeasts. Drug combination assays were performed using the checkerboard microdilution method (Pillai et al., 2005). The interactions between tacrolimus and azoles were assayed for *S. brasiliensis* and *S. schenckii* isolates. In addition, we determined the interaction between cyclosporine A and azoles for the reference strains (*S. brasiliensis* CBS 133021 and *S. schenckii* CBS 132984).

Drugs were diluted in RPMI 1640 medium supplemented with 2% glucose and buffered (to pH 7.2) with 0.165 M 3-(*N*-morpholine) propane sulfonic acid (MOPS). Activity assays were performed in 96-well microtiter plates containing 200 μl/well of medium with drugs and 0.5–1.5 × 10^5^ CFU/mL of yeasts, which were treated with drugs (alone or in combination) for 48 h, at 35°C, in a 5% CO_2_ atmosphere. Then, yeast growth was quantified by spectrophotometric readings at 490 or 492 nm, in a microtiter plate reader (SpectraMax Plus or EMax Plus, Molecular Devices). The percentage of yeast growth (G) relative to untreated controls was determined using to the formula: G = A × 100/C, where A is the absorbance value of treated wells and C is absorbance of untreated control wells. Minimum inhibitory concentration (MIC) values for drugs (as single agents, and within combinations) were defined as the lowest drug concentration that inhibited ≥50% of fungal growth relative to untreated controls.

For monotherapy assays, the following final concentrations of drugs were tested: 0.001 to 16 mg/L for itraconazole; 0.03 to 64 mg/L for fluconazole; 0.008 to 16 mg/L for tacrolimus; and 0.125 to 16 mg/L for cyclosporine A, in twofold serial dilutions. For combination assays, drugs were tested at concentrations up to the MIC determined in monotherapy assays, and MIC values were determined for each drug within combination assays. In addition, a fractional inhibitory concentration index (FICI) was calculated for each drug combination, using the equation: FICI = (MICa in combination/MICa alone) + (MICb in combination/MICb alone), where a and b are the drugs used in the combination assay (Pillai et al., 2005). Interactions were considered synergistic if FICI ≤ 0.5 (Odds, 2003). Results correspond to the most frequent value obtained in at least two independent experiments.

### Flow Cytometry Analysis

*Sporothrix brasiliensis* CBS 133021 and *S. schenckii* CBS 132984 yeast cells were left untreated or treated for 48 h with 0.5 mg/L tacrolimus, 0.01 mg/L itraconazole, or 1 mg/L fluconazole (alone or in combination). Then, cells were washed three times in PBS, incubated with 10 μM of BODIPY 496/503 (Molecular Probes^TM^, United States) for 45 min (at room temperature and in the dark), washed in PBS, fixed in 2% formaldehyde in PBS, and washed again. Samples were analyzed in a BD Accuri^TM^ C6 flow cytometer (BD Bioscience, United States), by counting 10000 events per sample, and data were analyzed using the BD Accuri C6 software. Results are representative of three independent experiments, performed in duplicates.

### Statistical Analysis

Statistical analyses were performed using the GraphPad Prism 5.0 software, by one-way ANOVA (with Dunnett’s *post hoc* test), to compare the effects of drugs (alone and in combination) on yeast growth, and to compare the accumulation of neutral lipids (as assessed by BODIPY 496/503 labeling) after treatment. A 5% significance level was adopted (*p* < 0.05). Mann–Whitney’s test (*t*-Student’s test) was also used to analyze differences in drug susceptibility between human- and feline-born *S. brasiliensis* isolates.

## Results

### Calcineurin Inhibitors Are Effective against *S. brasiliensis* and *S. schenckii* Yeasts

Calcineurin inhibitors potentiate the effect of azole therapy *in vitro*, against a variety of major fungal pathogens, including *Candida* species, *C. neoformans*, and *A. fumigatus*. Nevertheless, the effects of these drugs against sporotrichosis agents had not been tested previously. In the present study, we evaluated calcineurin inhibitors tacrolimus and cyclosporine A – as a monotherapy and in drug combinations – against reference strains *S. brasiliensis* CBS 133021 and *S. schenckii* CBS 132984. In drug activity assays, we used the yeast form of the pathogen, since this form is present during infection in the mammalian host. Tacrolimus inhibited fungal growth of *S. brasiliensis* CBS 133021 and *S. schenckii* CBS 132984 yeasts, with MIC values of 1 and 2 mg/L, respectively, while cyclosporine A inhibited yeast growth at 1 mg/L. MIC values of calcineurin inhibitors were considerably lower than the MIC values obtained for fluconazole (≥16 mg/L). In contrast, itraconazole inhibited yeast growth at lower concentrations than tacrolimus, cyclosporine A, and fluconazole (MIC = 0.125 and 0.25 mg/L, *S. brasiliensis* and *S. schenckii*, respectively) (**Table 2**).

**Table 2:** Antifungal activity of tacrolimus, cyclosporine A, itraconazole, and fluconazole – as monotherapy and in their most effective combination – against *S. brasiliensis* and *S. schenckii* reference isolates.

	MIC^a^ (mg/L)							
	
	TAC^b^	CYC^c^	ITC^d^	FLC^e^	TAC/ITC (FICI^f^)	TAC/FLC (FICI^f^)	CYC/ITC (FICI^f^)	CYC/FLC (FICI^f^)
*S. brasiliensis* CBS 133021	1	1	0.125	16	0.25/0.002 (0.27)	0.25/0.25 (0.27)	0.125/0.125 (1.13)	0.125/16 (1.13)
*S. schenckii* CBS 132984	2	1	0.25	32	0.25/0.001 (0.13)	0.25/0.125 (0.13)	0.125/0.25 (1.13)	0.125/32 (1.13)


*^a^MIC, minimum inhibitory concentration, defined as the concentration that inhibited ≥50% of yeast growth (shown as the median of two or more independent experiments); ^b^TAC, tacrolimus; ^c^CYC, cyclosporine A; ^d^ITC, itraconazole; ^e^FLC, fluconazole; ^f^FICI, fractional inhibitory concentration index = (MIC_*x*_ in combination/MIC_*x*_ alone) + (MIC_*y*_ in combination/MIC_*y*_ alone, where x is tacrolimus or cyclosporine A; and y is itraconazole or fluconazole. Interactions were considered synergistic if FICI ≤ 0.5.*

### Tacrolimus Increases the Antifungal Activities of Itraconazole and Fluconazole toward *Sporothrix* Yeasts

**FIGURE 1:**
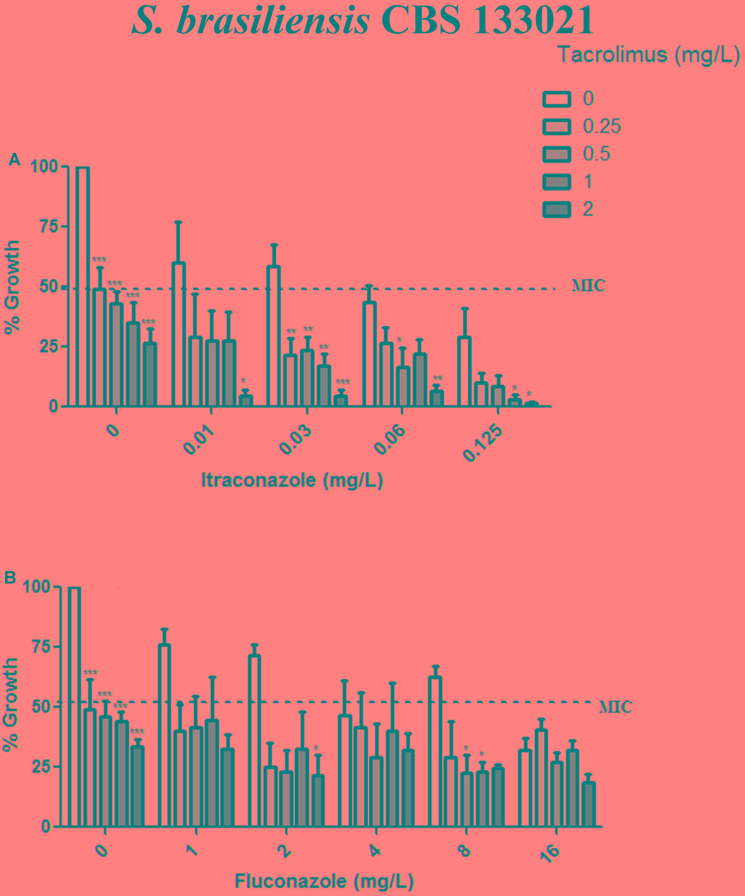
Effect of tacrolimus, alone or in combination with itraconazole or fluconazole, against the *Sporothrix brasiliensis* reference strain CBS 133021. **(A,B)** Yeast growth in the presence of tacrolimus combined with itraconazole **(A)** or fluconazole **(B)**. In combination assays, agents were tested by the checkerboard method, at concentrations up to the MIC obtained in monotherapy assays. Tacrolimus increased the inhibitory activity of itraconazole, in a dose-dependent manner. Data represent mean ± SEM of three independent experiments. ^∗^*p* < 0.05, ^∗∗^*p* < 0.001, ^∗∗∗^*p* < 0.0001 vs. treatment with the azole only, at the same concentration used in the combination assay (by one-way ANOVA and Dunnett’s test). MIC was defined as the concentration that inhibited ≥50% of yeast growth.

**FIGURE 2:**
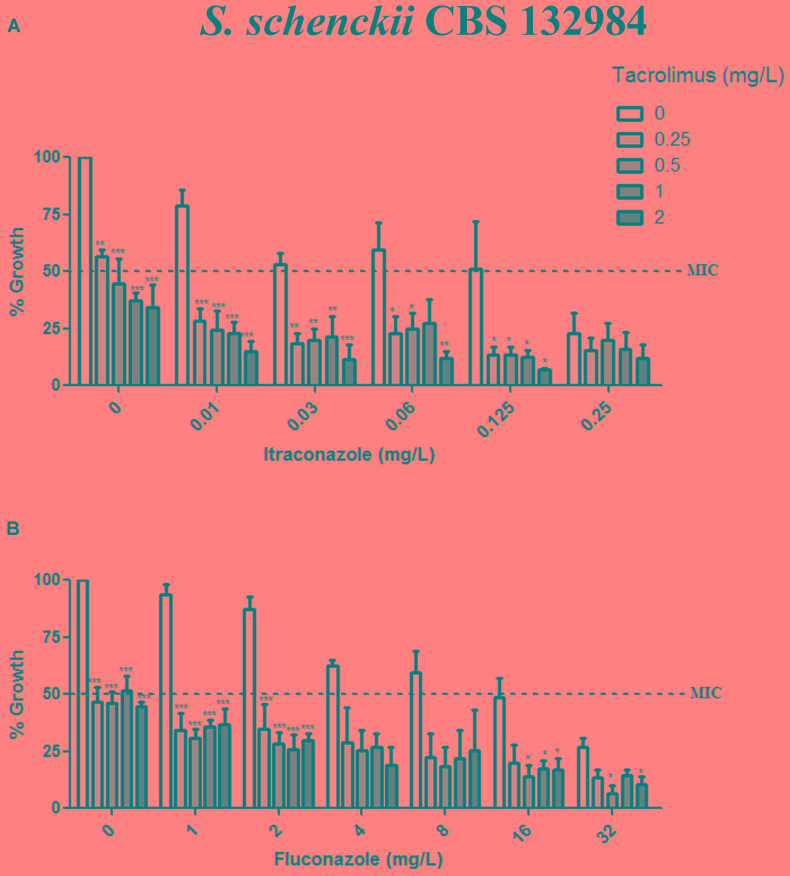
Effect of tacrolimus, alone or in combination with itraconazole or fluconazole, against the *Sporothrix schenckii* reference strain CBS 132984. **(A,B)** Yeast growth in the presence of tacrolimus combined with itraconazole **(A)** or fluconazole **(B)**. In combination assays, agents were tested by the checkerboard method, at concentrations up to the MIC obtained in monotherapy assays. Tacrolimus increased the inhibitory activity of both azoles, in a dose-dependent manner. Data represent mean ± SEM of three independent experiments. ^∗^*p* < 0.05, ^∗∗^*p* < 0.001, ^∗∗∗^*p* < 0.0001 vs. treatment with the azole only, at the same concentration used in the combination assay (by one-way ANOVA and Dunnett’s test). MIC was defined as the concentration that inhibited ≥50% of yeast growth.

Given than calcineurin inhibitors potentiates the effect of azoles against a variety of fungal pathogens, we tested whether tacrolimus and cyclosporine A would have a synergistic effect against *S. brasiliensis* and *S. schencki* when used in combination with itraconazole or fluconazole. When tacrolimus was combined with itraconazole or fluconazole (at maximum concentrations equal to MIC determined in monotherapy assays) the concentrations of individual drugs required to inhibited yeast growth were considerably lower than those required in monotherapy assays. The inhibitory activities of tacrolimus, itraconazole, and fluconazole were dose-dependent, whether these agents were used as monotherapy or in combination (**Figures 1**, **2**). According to FICI values, the effect of tracolimus combinations was synergistic (FICI ≤ 0.5), while cyclosporine A not exhibited synergistic effect when combined with itraconazole or fluconazole against *S. brasiliensis* CBS 133021 and *S. schenckii* CBS 132984 yeasts (**Table 2**). Thus, we used tacrolimus as the calcineurin inhibitor of choice in all further experiments.

### Exposure to Tacrolimus and Itraconazole Increased Neutral Lipid Accumulation in *S. brasiliensis* Yeasts

**FIGURE 3:**
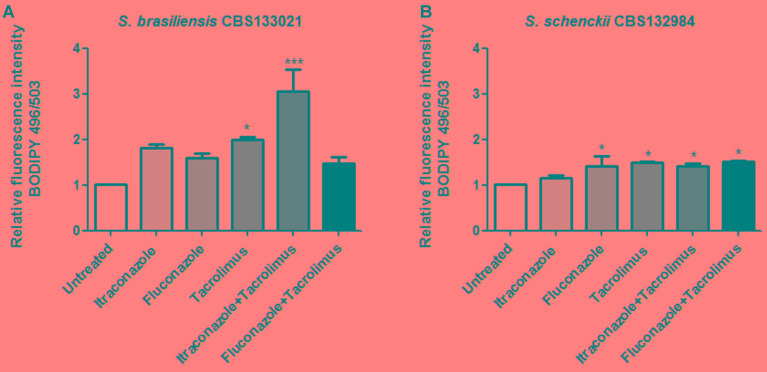
Effect of tacrolimus, itraconazole, and fluconazole – as monotherapy, or in synergistic combinations – on the levels of neutral lipids in *Sporothrix brasiliensis* CBS 133021 **(A)** and *S. schenckii* CBS 132984 **(B)**. Yeast cells were treated with drugs for 48 h, stained with BODIPY 493/503 and then analyzed by flow cytometry. The following drug concentrations were used: 0.5 mg/L tacrolimus, 0.01 mg/L itraconazole, and 1 mg/L fluconazole. The combination of tacrolimus with itraconazole increased considerably the levels of neutral lipids in *S. brasiliensis* yeast cells, relative to the untreated control. Data represent mean ± SEM values of three independent experiments. ^∗^*p* < 0.05, ^∗∗∗^*p* < 0.001 vs. untreated (by one-way ANOVA with Dunnett’s test).

Itraconazole and fluconazole inhibit ergosterol biosynthesis and induce the intracellular accumulation of neutral lipids, representing intermediary sterols from incomplete ergosterol biosynthesis (Robbins et al., 2016). Thus, we assessed whether the increased activity of itraconazole and fluconazole in combination with tacrolimus was associated with increase in the levels of neutral lipids. *S. brasiliensis* and *S. schenckii* yeasts were treated with synergistic combinations of drugs (0.5 mg/L tacrolimus and 0.01 mg/L itraconazole or 1 mg/L fluconazole), and then cells were stained with the fluorescent probe BODIPY 496/503, to detect neutral lipids (**Figure 3**). Itraconazole alone did not induced neutral lipid accumulation in either of the *Sporothrix* species tested here (**Figures 3A,B**). In contrast, treatment with tacrolimus alone led to a statistically significant increase in the neutral lipid levels in both *Sporothrix* species, and this effect was potentiated by the combination with itraconazole, mainly in *S. brasiliensis* yeasts (**Figure 3A**). Treatment with fluconazole alone increased the neutral lipid levels in *S. schenckii* yeasts only; however, this effect was relatively modest and was not potentiated by the combination with tacrolimus (**Figure 3B**). Overall, the combination of itraconazole and tacrolimus led to the highest levels of neutral lipid accumulation, in *S. brasiliensis* yeasts (**Figure 3A**).

### Combinations of Tacrolimus and Azoles Were Synergistic against Feline-Borne *S. brasiliensis* Isolates

In light of the potent effect of tacrolimus and azoles combinations against the reference strains, we evaluated the interaction between tacrolimus and azoles against 10 disease isolates of *S. brasiliensis* (five from cats and five from humans) and five clinical isolates of *S. schenckii* (**Table 3**). Clinical isolates of *S. braziliensis* from patients with sporotrichosis (from the Rio de Janeiro state, Brazil) were considerably more sensitive to tacrolimus as monotherapy than feline-borne isolates, with mean MIC values of 0.23 and 2.2 mg/L (for clinical and feline-borne isolates, respectively; *p* = 0.0112) (**Table 3**). Tacrolimus was as effective as itraconazole against *S. braziliensis* clinical isolates (*p* = 0.5186). Clinical isolates of *S. schenckii* were also high sensitive to tacrolimus as monotherapy (MIC value of 0.112 mg/L), as well as to itraconazole (MIC value of 0.05 mg/L) (**Table 3**). However, combinations of tacrolimus and itraconazole or fluconazole have synergistic antifungal effect (FICI ≤ 0.5) only for feline-borne *S. brasiliensis* isolates (**Table 3**).

**Table 3:** Antifungal activity of tacrolimus, itraconazole, and fluconazole – as monotherapy and in their most effective combination – against *S. brasiliensis* and *S. schenckii* isolates.

		MIC^a^ (mg/L)				
		
		TAC^b^	ITC^c^	FLC^d^	TAC/ITC (FICI^e^)	TAC/FLC (FICI^e^)
***S. brasiliensis***	**Clinical isolates**					
	B204	0.25	0.25	32	0.125/0.25 (1.5)	0.03/32 (1.12)
	B428	0.5	0.125	64	0.03/0.125 (1.06)	64/0.03 (1.06)
	B435	0.125	0.125	32	0.03/0.06 (0.72)	0.03/16 (0.74)
	B735	0.125	0.125	16	0.03/0.125 (1.24)	0.03/8 (0.62)
	B972	0.125	0.125	32	0.03/0.125 (1.24)	0.03/32 (1.24)
	Mean values	0.23	0.15	35.2	-	-
	**Feline-borne isolates**					
	Ss 53	2	0.06	4	0.125/0.001 (0.08)	0.03/0.03 (0.02)
	Ss 172	4	0.06	32	0.06/0.001 (0.03)	0.125/0.125 (0.04)
	Ss 245	2	0.25	8	0.125/0.001 (0.07)	0.125/0.03 (0.07)
	Ss 246	1	0.125	32	0.06/0.125 (1.06)	0.06/32 (1.06)
	Ss 294	2	0.125	32	0.125/0.001 (0.07)	0.125/2 (0.1)
	Mean values	2.2	0.12	21.6	-	-

***S. schenckii***	**Clinical isolates**					
	Ss 03	0.125	0.06	16	0.06/0.03 (0.98)	0.008/16 (1.06)
	Ss 42	0.125	0.06	32	0.015/0.3 (0.62)	0.008/32 (1.06)
	Ss 73	0.125	0.06	32	0.008/0.06 (1.06)	0.008/32 (1.06)
	Ss 75	0.06	0.06	32	0.008/0.06 (1.13)	0.008/32 (1.13)
	Ss 110	0.125	0.03	16	0.06/0.015 (0.98)	0.008/8 (0.6)
	Mean values	0.112	0.05	25.6	-	-


*^a^MIC, minimum inhibitory concentration, defined as the concentration that inhibited ≥50% of yeast growth (shown as the median of two or more independent experiments); ^b^TAC, tacrolimus; ^c^ITC, itraconazole; ^d^FLC, fluconazole; ^e^FICI, fractional inhibitory concentration index = (MIC_*x*_ in combination/MIC_*x*_ alone) + (MIC_*y*_ in combination/MIC_*y*_ alone, where x is tacrolimus and y is itraconazole or fluconazole. Interactions were considered synergistic if FICI ≤ 0.5.*

## Discussion

The incidence of sporotrichosis in Brazil is high compared with that observed in other countries, and the most recent outbreaks in the state of Rio de Janeiro (caused by inoculation of yeasts from infected cats) have highlighted the urgent need to expand the (thus far) limited therapeutic ‘arsenal’ against this disease. Here, we demonstrated that the calcineurin inhibitors tacrolimus and cyclosporine A are active *in vitro* (as monotherapy) against *S. brasiliensis* and *S. schenckii* yeasts. Importantly, tacrolimus also increased the antifungal activity of itraconazole and fluconazole, the current first- and second-line agents, respectively, against *S. brasiliensis* feline-borne isolates. The increased effect of combination therapy with tacrolimus is particularly important for fluconazole, which has relatively low antifungal activity against *Sporothrix* spp. (Marimon et al., 2008; Ottonelli-Stopiglia et al., 2014; Rodrigues et al., 2014a), but represents an alternative to the costly, lengthy, and comparatively more toxic therapy with itraconazole.

While tacrolimus had considerable antifungal activity against *S. brasiliensis* and *S. schenckii* yeasts from reference isolates (with MIC values of 1 and 2 mg/L, respectively), it was very active against clinical isolates of *S. brasiliensis* and *S. chenckii* (MIC value of 0.23 and 0.112 mg/L, respectively), which represents an inhibitory efficacy comparable to that of itraconazole. *Sporothrix* spp. was more susceptible to tacrolimus than other yeasts or filamentous pathogenic fungi (Del Poeta et al., 2000; Sun et al., 2008; Li et al., 2014; Gao and Sun, 2015). This higher sensitivity could be due to the dimorphic nature of *Sporothrix* spp., where dimorphism is required for the infection cycle. In *Mucor circinelloides*, treatment with tacrolimus interferes with dimorphism leading to a high reduction in growth, due to calcineurin inhibition (Lee et al., 2013). In addition, the synergistic effect between tacrolimus and azoles against *Sporothrix* spp. confirms the effect described for other fungi of medical relevance (Del Poeta et al., 2000; Sun et al., 2008; Uppuluri et al., 2008; Li et al., 2014; Denardi et al., 2015; Gao and Sun, 2015).

The evaluation of neutral lipid levels – which showed pronounced neutral lipid accumulation after treatment with tacrolimus and itraconazole, in *S. brasiliensis* CBS 133021 – was in agreement with the activity assays data showing that *S. brasiliensis* yeasts were more sensitive to tacrolimus combined with itraconazole than combined with fluconazole. The neutral lipid accumulation and antifungal activity data also show that *S. brasiliensis* CBS 133021 yeasts (a strain originally isolated from feline sporotrichosis) are more sensitive to tacrolimus (alone and in combination therapy) than *S. schenckii* CBS 132984 yeasts. Interestingly, despite clinical isolates of *S. brasiliensis* and *S. schenckii* showed higher sensitivity to tacrolimus as monotherapy than feline-borne isolates, treatment with tacrolimus only increased the antifungal activity (i.e., decreased the MIC) of azoles against feline-borne isolates of *S. brasiliensis*, showing synergistic effect (FICI ≤ 0.5).

A small intra-species variation has been reported in *S. brasiliensis* species (Oliveira et al., 2015). Because the different isolates are genetically very similar, it was proposed that the species be a clone (Rodrigues et al., 2014b). Nevertheless, we observed differences in the drug susceptibility between individual isolates, and also between the groups of feline-borne and clinical isolates, which highlights the existence of clinically relevant heterogeneity within the *S. brasiliensis* species. This heterogeneity could be related to the intra-species genetic variation and/or differences in gene expression. Almeida-Paes et al. (2017) reported that *S. brasiliensis* isolates from human and cats with sporotrichosis had similar sensibility to azoles, but exhibited differences to amphotericin B and terbinafine susceptibilities (Almeida-Paes et al., 2017). The lower sensitivity of feline-borne isolates to tacrolimus as monotherapy may stem from an adaptation of fungal isolates to the higher body temperature encountered in cats (compared with that found in humans). Under higher temperatures, feline-borne isolates may have higher calcineurin activation (compared to human isolates) because calcineurin turnover is regulated by interaction with Hsp90, and without stress conditions Hsp90 inhibits calcineurin activity (Stie and Fox, 2008). However, exposure to stress factors promotes the dissociation of Hsp90, activating calcineurin (Stie and Fox, 2008).

The main mechanism of action of tacrolimus in eukaryotic cells is the inhibition of calcineurin activity (Tompson et al., 1995). However, in the dimorphic fungus *Paracoccidioides brasiliensis*, tacrolimus does not inhibit calcineurin activity; rather, its effects on the fungus are due to efflux pump inhibition (Campos et al., 2008). Similarly, in *C. neoformans*, the synergistic effect of the tacrolimus/fluconazole combination is due to efflux pump inhibition, and not to calcineurin inhibition (Del Poeta et al., 2000). In the present study, treatment with tacrolimus alone increased neutral lipid levels (compared with the untreated yeasts). These results suggest that tacrolimus might inhibits ABC transporters in *Sporothrix* spp., since these molecules also act on lipid translocation in the lipid bilayer (Cannon et al., 2009), and inhibition of their activity could disturb lipid transport and promote intracellular neutral lipid accumulation.

Cyclosporine A, another calcineurin inhibitor, inhibited yeast growth at 1 mg/L; however, it did not have a synergistic effect when combined with itraconazole or fluconazole, against the *S. brasiliensis* and *S. schenckii* reference isolates tested here. These results support the notion that tracolimus might inhibits ABC transporter in *Sporothrix* spp., which counteracts efflux-pump mediated drug resistance, being the main factor responsible for the synergy between tacrolimus and azoles in this study. The apparent influence of efflux pumps on the profile of sensitivity to azoles needs to be investigated further, since itraconazole is the main antifungal used in the treatment of sporotrichosis. Still, the inhibitory activity of tacrolimus monotherapy could be due its action on calcineurin, and further studies are required to confirm the mechanism of action of tacrolimus against *Sporothrix* spp.

In summary, our results show that drug tacrolimus has promising antifungal activity against sporotrichosis agents, and also increases the activity of the current anti-sporotrichosis therapy (itraconazole and fluconazole) in combination assays against *S. brasiliensis* feline-borne isolates, *in vitro*. The synergistic activity of tacrolimus may be due to efflux pump (rather than calcineurin) inhibition, since it is not present in a different calcineurin inhibitor (cyclosporine A). Further studies on the mechanism of action of tacrolimus in *Sporothrix* spp. should aid in the development related molecules with increased selectivity for fungi, and increased potential for clinical use against sporotrichosis.

## Author Contributions

LB-S carried out all the experiments and drafted the manuscript. LRS, JR, AF-P, and SR designed and coordinated the study. AR and ZC provided *S. brasiliensis* feline-borne isolates and *S. schenckii* isolates used in this work and helped to draft the manuscript. All authors read, contributed, and approved the final manuscript.

## Conflict of Interest Statement

The authors declare that the research was conducted in the absence of any commercial or financial relationships that could be construed as a potential conflict of interest.
